# Regulation of Paracellular Fluxes of Amino Acids by Claudin-8 in Normal Mouse Intestinal MCE301 Cells

**DOI:** 10.3390/nu15061346

**Published:** 2023-03-10

**Authors:** Ema Okamoto, Shunsuke Matsuda, Yuta Yoshino, Yoshifumi Morikawa, Koichi Suenami, Yoshiaki Tabuchi, Toshiyuki Matsunaga, Akira Ikari

**Affiliations:** 1 Laboratory of Biochemistry, Department of Biopharmaceutical Sciences, Gifu Pharmaceutical University, Gifu 501-1196, Japan; 2 Forensic Science Laboratory, Gifu Prefectural Police Headquarters, Gifu 500-8501, Japan; 3 Life Science Research Center, University of Toyama, Toyama 930-0194, Japan; 4 Laboratory of Bioinformatics, Gifu Pharmaceutical University, Gifu 502-8585, Japan

**Keywords:** aging, amino acid, claudin-8, paracellular flux

## Abstract

The ingested proteins are catabolized to di/tri-peptides and amino acids (AAs), which are absorbed through various transporters in the small intestinal and colonic epithelial cells. Tight junctions (TJs) are formed between neighboring cells and restrict paracellular fluxes to mineral ions and aqueous molecules. However, it is unknown whether the TJs are implicated in the control of paracellular fluxes to AAs. The paracellular permeability is controlled by claudins (CLDNs), which comprise a family of over 20 members. Here, we found that CLDN8 expression is decreased by AAs deprivation in normal mouse colon-derived MCE301 cells. The reporter activity of CLDN8 was not significantly changed by AAs deprivation, whereas the stability of CLDN8 protein was decreased. MicroRNA analysis showed that AAs deprivation increases the expression of miR-153-5p which targets CLDN8. The AAs deprivation-induced decline of CLDN8 expression was reversed by a miR-153-5p inhibitor. The CLDN8 silencing enhanced the paracellular fluxes to AAs, especially middle molecular size AAs. The expression levels of colonic CLDN8 and miR-153-5p in aged mice were lower and higher than those in young mice, respectively. We suggest that AAs deprivation downregulates CLDN8-dependent barrier function, mediated by the elevation of miR-153-5p expression in the colon, in order to enhance the AAs absorption.

## 1. Introduction

Dietary proteins are digested into di/tri-peptides and free amino acids (AAs) in the stomach and small intestine. About 90% of the dietary proteins are absorbed in the small intestine, and the remaining 10% reach the colon [1]. In addition, the AAs released from endogenous proteins can enter the colon [2]. Among the 20 L-type AAs, 9 AAs are considered nutritionally essential and must be supplied by the diet because they cannot be synthesized within the body. AAs are not considered to be absorbed in the colon under normal conditions in days gone by. However, several reports indicate that substantial amounts of AAs can be absorbed in the colon [3,4]. The body contents of AAs must be modulated by the absorption from the small intestine and colon, but the absorption rate of AAs declines with age in rats [5], mice [6], and humans [7]. Before transport into the portal vein, AAs are metabolized in the intestinal epithelial cells, which are used for the construction of an intestinal structure, renewal of epithelium, cellular metabolism, and growth [8]. In addition, the absorbed AAs are made available for protein synthesis, conversion to other AAs, energy synthesis, and so on. Therefore, the lack of AAs can lead to age-related diseases such as physical frailty and sarcopenia [9].

Many kinds of AA transporters are present in the epithelial cells of the small intestine and colon [10], which may promote the influx of AAs from the lumen to the blood. AAs can be taken up into the cells via electroneutral or electrogenic transport systems [11]. In the small intestine, various transporters have been identified for the absorption of neutral AAs (B^0^AT1; ASCT2), cationic AAs (blastocyst neutral and cationic amino acid transporter, b^0,+^AT), glycine and proline (PAT1), neutral and basic AAs (SLC3A1), anionic AAs (EAAT3), and so on. Some of them are also present in the colon [1]. The expression levels of EAAT3, ASCT2, and rBAT in the middle jejunum are downregulated in the pigs fed with low-protein diets [12]. The expression levels of EAAT3 in the rabbit jejunum show a positive correlation with food protein contents [13]. In addition, the expression levels of most AA transporters are changed by the stage of development. The dynamic change in expression according to diets and aging may be necessary to maintain the balance of AAs in the body.

Intestinal epithelial cells form tight junctions (TJs) at the most apical region of the lateral membrane between two adjacent cells [14]. The TJs restrict the paracellular transports of mineral ions, polar solutes, and macromolecules. In addition, the TJs provide a physical barrier to the lateral diffusion of plasma membrane proteins and lipids. Several components including transmembrane, peripheral, and cytoskeletal proteins are involved in the formation of TJs. The peripheral proteins including zonula occludens-1 (ZO-1) and ZO-2 function as scaffolding and signaling molecules. So far, several transmembrane proteins of the TJs have been identified such as claudins (CLDNs), occludin (OCLN), and junctional adhesion molecules. CLDNs are four-transmembrane proteins with a molecular weight of about 23 kDa and comprise a family of over 20 members in a mammal [15,16,17]. Each CLDN subtype is expressed in a tissue-specific manner and confers different properties on paracellular barrier functions. CLDN1, 2, 7, 12, and 15 are commonly expressed in both the small intestine and colon. In contrast, CLDN5 and 18 are enriched in the small intestine, whereas CLDN3, 4, 8, and 13 are enriched in the colon. The experiments using knockout mice and knockdown cells show that CLDN2 and 15 form a cation-permeable pore, CLDN10a and 17 form an anion-pore, and CLDN4, 8, and 14 form a charge-selective barrier [18,19]. However, it is unknown whether AAs are transported via a paracellular pathway and which CLDN subtype is directly implicated in the regulation of paracellular AA fluxes.

In the current study, we found that AAs deprivation induces the reduction in *CLDN8* mRNA expression in normal mouse colon-derived MCE301 cells. Therefore, the expression and localization of CLDN8 protein were investigated by Western blotting and immunocytochemistry, respectively. The paracellular fluxes to mineral ions and AAs were examined by volt-ohm meter and L-AA colorimetric assay, respectively. The AAs composition was determined by liquid chromatography–mass spectrometry (LC–MS) analysis.

## 2. Materials and Methods

### 2.1. Materials

The primary antibodies used in the studies were purchased from the sources listed in Table 1. Actinomycin D, chloroquine, 4′,6-diamidino-2-phenylindole (DAPI), dorsomorphin, 3-methyladenine (3MA), and MHY1485 were obtained from Focus Biomolecules (Plymouth Meeting, PA, USA), Santa Cruz Biotechnology (Santa Cruz, CA, USA), Dojindo Laboratories (Kumamoto, Japan), Fujifilm Wako Pure Chemical Industries (Osaka, Japan), Cayman Chemical Company (Ann Arbor, MI, USA), Tokyo Kasei Kogyo (Tokyo Japan), respectively. A miR-153-5p inhibitor was generated by Integrated DNA Technologies (mmmu-miR-153-5p, Coralville, IA, USA). All other chemicals were of the purest grade available.

### 2.2. Animals

Male C57BL/6 young (6 weeks, *n* = 5) and aged (49 weeks, *n* = 5) mice were purchased from Japan SLC (Hamamatsu, Japan), and were namely the young and aged groups, respectively. The experiments were carried out after 7 days of acclimation. All the experiments using animals were approved by the Animal Care and Use Committee of the Gifu Pharmaceutical University (No. 2021-081) and executed in adherence with the Guidelines and Regulations for the Care and Use of Experimental Animals by the Gifu Pharmaceutical University. The mice were fed with a normal diet and distilled water *ad libitum*. The segments of the middle to distal colon were isolated and stored in RNA stabilizer.

### 2.3. Cell Culture, Transfection, and Reporter Assay

MCE301 cells were established from a normal mouse colon [20]. The cells were cultured as described previously [21]. After culturing for 14 days, the cells were used for analyses of real-time polymerase chain reaction (PCR), Western blotting, immunocytochemistry, and transwell fluxes to mineral ions and AAs. The culture media without AAs were used to control the concentration of AAs. The reporter plasmids of mouse CLDN8/pEZX-PG02 (GeneCopoeia, Rockville, MD, USA) and pSEAP2 control vector (Takara Bio, Shiga, Japan) were introduced into the cells using Lipofectamine 2000 (Thermo Fisher Scientific) in line with the instruction manual. The reporter activity was measured using a Secrete-Pair Dual Luminescence Assay Kit (GeneCopoeia). The siRNAs for negative control and CLDN8 were purchased from Sigma-Aldrich. These siRNAs were introduced into the cells using Lipofectamine RNAiMAX Transfection Reagent (Thermo Fisher Scientific).

### 2.4. Real-Time PCR

The isolation of total RNA and real-time reverse transcription PCR were conducted as described previously [21]. The primer pairs of TJ components are listed in Table 2. The mRNA levels were compensated by β-actin. For the assay of microRNA (miRNA) expression, reverse transcription and real-time PCR were conducted using a Mir-X miRNA qRT-PCR SYBR Kit (Takara Bio). The primer pairs of miRNAs are listed in Table 3.

### 2.5. Western Blot Analysis

The preparation of cell lysates, sodium dodecyl sulfate-polyacrylamide gel electrophoresis, and immunoblotting were conducted as described previously [21]. β-Actin was used for a loading control and all samples were standardized by β-actin. The band density of each protein was analyzed using ImageJ software (National Institute of Health, Bethesda, MD, USA).

### 2.6. Immunocytochemistry

Cells were seeded at a density of 5 × 10^4^ cells onto a 35 mm culture dish with a cover glass on the bottom. Both CLDN8 and ZO-1 were stained with each primary antibody and then stained with secondary antibodies conjugated to Alexa Fluor 555 and 488, respectively. The nuclei were visualized by DAPI. The cellular distribution of CLDN8 and ZO-1 was visualized using a confocal laser scanning microscope (LSM700, Carl Zeiss, Jena, Germany).

### 2.7. Paracellular Barrier to Electrolyte Ions and AAs

Cells were seeded at a density of 5 × 10^3^ cells onto 0.4 μm pore polyester membrane inserts in the transwell plates (Corning Incorporated, Corning, NY, USA). The fluxes to mineral ions via TJs were estimated by transepithelial electrical resistance (TER) with a volt ohm-meter (Millipore). For measurement of AA fluxes, the solutions of upper and lower chambers were replaced with 100% and 0% AAs media, respectively. To avoid the involvement of AA transporters, the cells were treated for 1 h at 4 °C. Then, the solution in the lower chamber was collected. The AAs concentration was measured using an L-Amino Acid Assay Kit (Colorimetric) (Cell Biolabs, San Diego, CA, USA). For liquid chromatography/mass spectrometry (LC/MS) analysis, the aliquots (20 μL) were deproteinated by the addition of an equal volume of sulfosalicylic acid solution (5% final concentration), and then they were centrifuged at 12,000× *g* for 15 min. The supernatants were used for the measurement of AA levels. The analysis took place using a Nexera system coupled to an LCMS-9030 mass spectrometer (Shimadzu, Kyoto, Japan) and an Intrada Amino Acid column (Imtakt, Kyoto, Japan; 150 mm × 2 mm, 3 μm particle size). The column was operated with a mobile phase consisting of acetonitrile/formic acid (100:0.3, *v*/*v*) (solvent A) and acetonitrile/100 mM ammonium formate (20:80, *v*/*v*) (solvent B). The elution was performed under the following conditions: isocratic conditions of 18% B were maintained for the first 6 min; gradient conditions increasing linearly to 45% B were achieved for 11 min; then, the gradient was changed to 100% B over 11–20 min; and finally, 100% B was held for 5 min at a flow rate of 0.3 mL/min. The mass spectra were acquired in positive ion mode using an electrospray ionization probe. LC/MS carried out quantitative measurement of the 19 AAs in the selected ion monitoring mode, and the protonated molecules ([M + H]^+^) monitored are shown in Table 4.

### 2.8. Statistic Analyses

Data are expressed as means ± S.E.M. Statistical analyses were performed using KaleidaGraph (version 4.5.1, Synergy Software, PA, USA) with *p* < 0.05 considered as statistically significant. Tukey’s multiple comparison test and Student’s *t* test were applied to the comparisons between multiple groups and two groups, respectively.

## 3. Results

### 3.1. Effects of AAs Deprivation on the Expression of TJs Components

**Figure 1:**
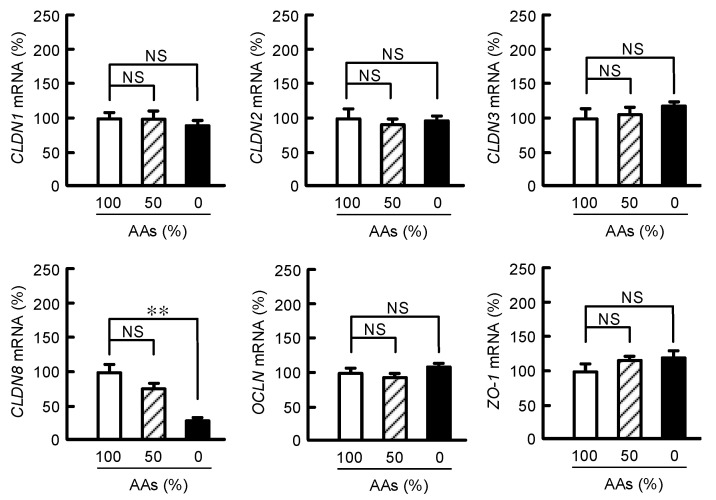
Effect of AAs deprivation on the expression levels of TJ components in MCE301 cells. Cells were treated with the media containing 100%, 50%, and 0% AAs for 6 h. The mRNA levels of TJ components were investigated by real-time PCR and were normalized by β-actin. Then, the expression levels were shown as a percentage of 100% AAs. *n* = 3–4. ** *p* < 0.01 and ^NS^ *p* > 0.05.

A number of CLDNs including CLDN1-3, OCLN, and ZO-1 were expressed in MCE301 cells (Figure 1). The mRNA levels of *CLDN1-3*, *OCLN*, and *ZO-1* were unchanged by AAs deprivation. In contrast, the *CLDN8* mRNA level was significantly decreased by AAs deprivation. These results indicate the possibility that CLDN8 may be involved in the regulation of AAs transport or metabolism.

### 3.2. Effects of AAs Deprivation on the Expression and Cellular Localization of CLDN8 Protein

**Figure 2:**
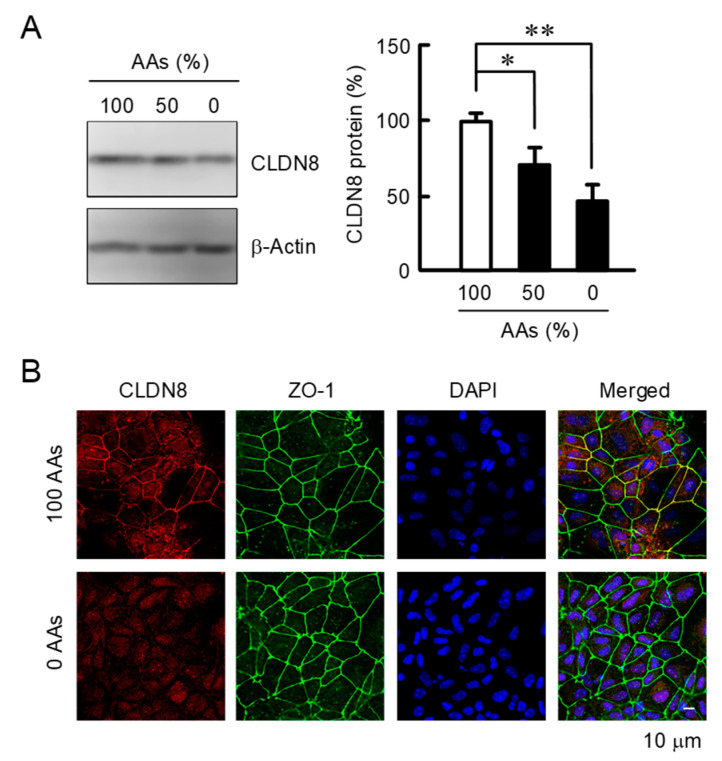
Effect of AAs deprivation on expression and cellular localization of CLDN8 protein. Cells were treated with the media containing 100%, 50%, and 0% AAs for 24 h. (**A**) The protein levels of CLDN8 were investigated by Western blotting and shown as a percentage of 100% AAs. (**B**) The distribution patterns of CLDN8 and ZO-1 were indicated by red and green fluorescence, respectively. The nuclei were stained with DAPI (blue) and the merged images are shown on the right. Scale bar represents 10 μm. *n* = 4. ** *p* < 0.01 and * *p* < 0.05.

The protein level of CLDN8 was significantly reduced by AAs deprivation (Figure 2A). There was good concordance between the real-time PCR and Western blot data. CLDN8 was seen in both the cytosol and adjacent to the lateral membrane under the normal AAs medium (Figure 2B). The distribution pattern at the lateral membrane coincided with ZO-1. AAs deprivation weakened the fluorescence intensity of CLDN8 without changing that of ZO-1. These data demonstrate that the mRNA and protein levels of CLDN8 are regulated by AAs concentration.

### 3.3. Regulatory Mechanism of AAs Deprivation-Induced Decline of CLDN8 Expression

**Figure 3:**
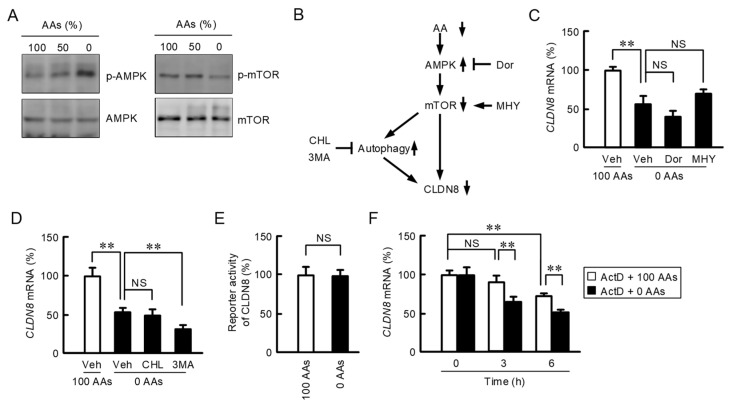
Intracellular signaling pathway in the control of CLDN8 expression. (**A**) Cells were treated with the media containing 100%, 50%, and 0% AAs for 1 h. The levels of p-AMPK, AMPK, p-mTOR, and mTOR were measured by Western blot analysis. (**B**) A putative scheme of the signal transduction of AAs deprivation. (**C**,**D**) The cells were treated with vehicle (Veh), 10 μM dorsomorphin (Dor), 10 μM MHY1485 (MHY), 20 μM chloroquine (CHL), and 3 mM 3MA in the media containing 100% or 0% AAs for 6 h. The expression levels of *CLDN8* mRNA were shown as a percentage of 100% AAs. (**E**) The cells transfected with CLDN8 reporter vector were treated with the media containing 100% or 0% AAs for 24 h. The reporter activity was shown as a percentage relative to 100% AAs. (**F**) The cells were treated with 10 μM actinomycin D (ActD) in the media containing 100% or 0% AAs for indicated periods. The expression levels of *CLDN8* mRNA were shown as a percentage of 0 h. *n* = 3–4. ** *p* < 0.01 and ^NS^ *p* > 0.05.

mTOR plays a central role in mediating AAs sensing, AAs transport, autophagy, and immunity [22]. The levels of p-AMPK were elevated by AAs deprivation, whereas those of mTOR were decreased (Figure 3A). These data demonstrate that the AMPK/mTOR pathway is activated by AAs deprivation in MCE301 cells. In order to elucidate the involvement of AMPK, mTOR, and autophagy events, we investigated the effects of inhibitors or activators shown in Figure 3B on CLDN8 expression. The AAs deprivation-induced decline of CLDN8 expression was not significantly inhibited by dorsomorphin, an AMPK inhibitor, and MHY1485, an mTOR activator (Figure 3C). These data demonstrate that the AMPK/mTOR pathway may not be implicated in the regulation of CLDN8 expression. In addition, the AAs deprivation-induced effect was not significantly suppressed by 3-MA and chloroquine (Figure 3D) which are autophagy inhibitors. 3-MA and chloroquine can inhibit the autophagy events at the early and late stages, respectively [23]. These data demonstrate that autophagy events may also not be implicated. The luciferase reporter gene assay showed that AAs deprivation may not be implicated in the regulation of transcriptional activity of CLDN8 (Figure 3E). In order to elucidate the effect of AAs deprivation on the mRNA stability of *CLDN8*, we performed an actinomycin D assay. In the presence of actinomycin D, the mRNA level of *CLDN8* diminished in a time-dependent manner, which was accelerated by AAs deprivation (Figure 3F). These results indicate that AAs deprivation may decrease the mRNA expression of *CLDN8*, mediated via the alteration of mRNA stability.

### 3.4. Involvement of miRNA-153-5p in the AAs Deprivation-Induced Decline of CLDN8 mRNA

**Figure 4:**
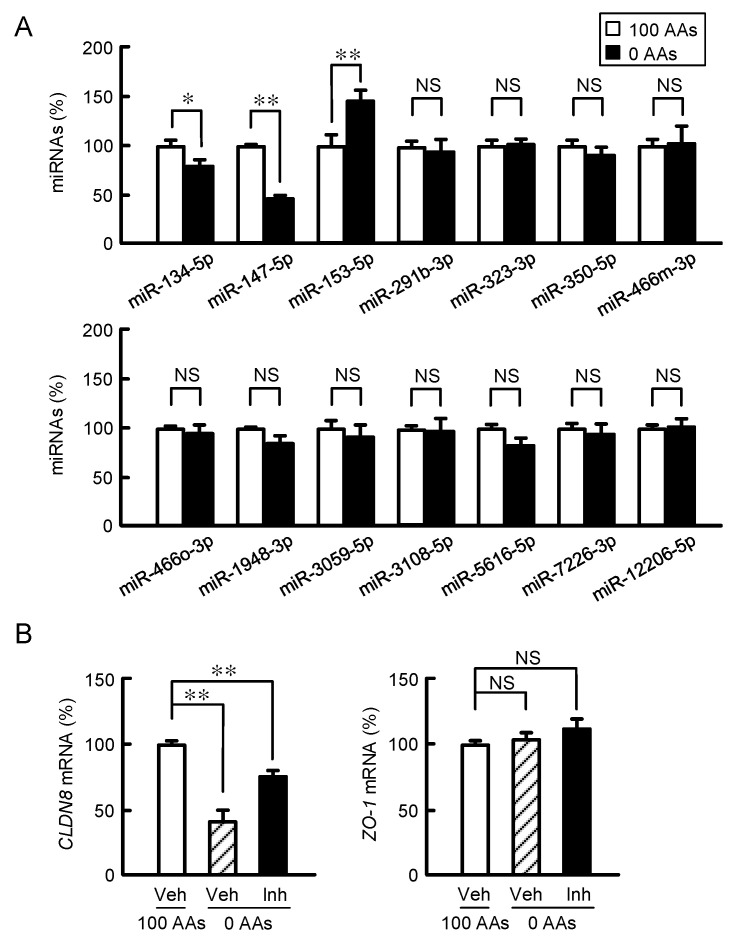
Involvement of miRNA in the regulation of CLDN8 expression. (**A**) Cells were treated with the media containing 100% and 0% AAs for 6 h. The miRNA levels were shown as a percentage of 100% AAs. (**B**) The cells were treated with vehicle (Veh) and miR-153-5p inhibitor (Inh) in the media containing 100% or 0% AAs for 6 h. The expression levels of *CLDN8* and *ZO-1* mRNAs were shown as a percentage of 100% AAs. *n* = 3–4. ** *p* < 0.01, * *p* < 0.05, and ^NS^ *p* > 0.05.

Computer analysis using the miRNA targets software MIRANDA predicted that miR-134-5p, miR-147-5p, miR-153-5p, miR-291b-3p, miR-323-3p, miR-350-5p, miR-466m-3p, miR-466o-3p, miR-1948-3p, miR-3059-5p, miR-3108-3p, miR-5616-3p, miR-7226-3p, and miR-12206-5p can target the 3′-untranslated region of *CLDN8* mRNA. The expression level of miR-153-5p was significantly increased by AAs deprivation (Figure 4A). In contrast, those of other miRNAs were unchanged or decreased by AAs deprivation. The treatment with miR-153-5p inhibitor blocked the AAs deprivation-induced decline of CLDN8 expression without affecting ZO-1 expression (Figure 4B). These data demonstrate that miR-153-5p may participate in the AAs deprivation-induced decline of *CLDN8* mRNA.

### 3.5. Effect of CLDN8 Expression on TER and Paracellular AA Fluxes

**Figure 5:**
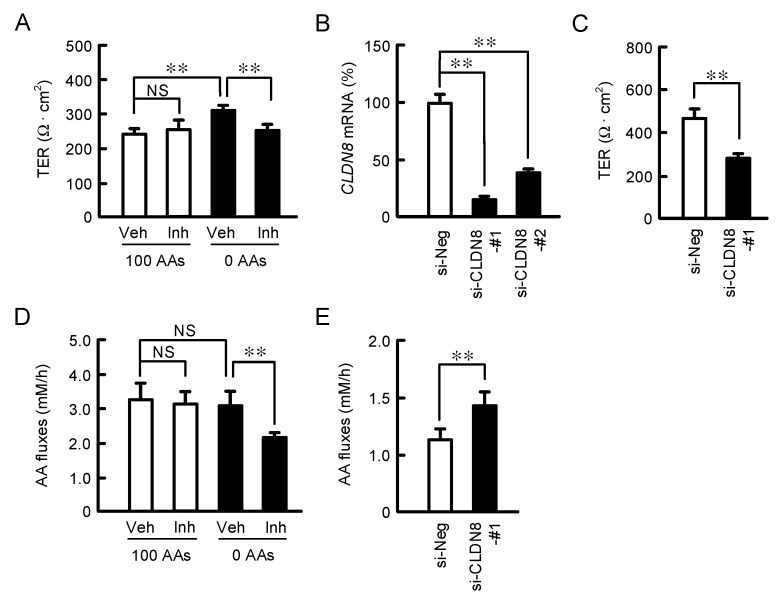
Effects of AAs deprivation and miRNA inhibitor on tight junctional barrier. (**A**,**D**) Cells were treated with vehicle (Veh) and miR-153-5p inhibitor (Inh) in the media containing 100% or 0% AAs for 24 h. TER was monitored by a volt-ohm meter. In the assay of AA fluxes, the solutions in the apical and basal chambers were replaced with the solutions containing 100% and 0% AAs, respectively. After treatment for 1 h at 4 °C, the solution of basal chamber was collected. AAs contents were measured using an ELISA kit. (**B**,**C**,**E**) The cells were transfected with negative (si-Neg) and CLDN8 siRNA (si-CLDN8-#1 and #2). The expression levels of *CLDN8* mRNAs were shown as a percentage of si-Neg. TER and AA fluxes were examined. *n* = 4–5. ** *p* < 0.01 and ^NS^ *p* > 0.05.

**Figure 6:**
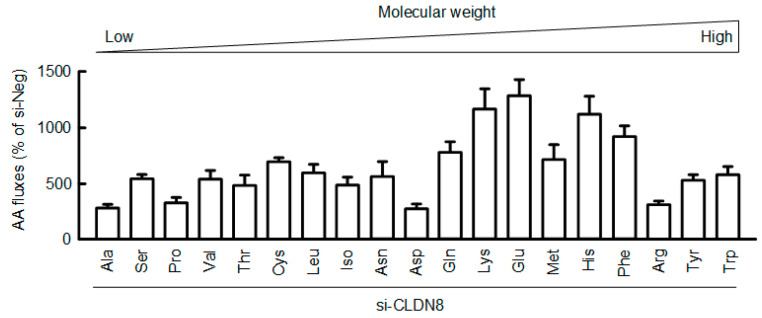
Effect of CLDN8 knockdown on paracellular fluxes of AAs. Negative (si-Neg) and CLDN8 siRNAs were introduced into the cells using a Lipofectamine RNAiMAX Transfection Reagent. In the assay of AA fluxes, the components of AAs in the basal chambers were measured by LC–MS. The contents of each AA in the cells transfected with CLDN8 siRNA were shown as a percentage of si-Neg. *n* = 5.

In order to elucidate the function of CLDN8, the paracellular permeability to mineral ions was estimated by TER. TER was increased by AAs deprivation (Figure 5A), which means the elevation of barrier function against mineral ions. The involvement of *miR-153-5p* was investigated using the miR-153-5p inhibitor, which binds to a target miRNA and inhibits its function. The effect of AAs deprivation was blocked by the miR-153-5p inhibitor. The silencing effects of two siRNAs (#1 and #2) for CLDN8 were checked by real-time PCR. The mRNA level of *CLDN8* was more potently suppressed by the siCLDN8-#1 (Figure 5B). Therefore, we decided to investigate the effect of siCLDN8-#1 on TER and AA fluxes. TER was significantly reduced by *CLDN8* silencing (Figure 5C). The AA fluxes were unchanged by AAs deprivation, but rescued by the miR-153-5p inhibitor (Figure 5D). The AA fluxes were significantly increased by *CLDN8* silencing (Figure 5E). These data demonstrate that CLDN8 may function as a barrier to paracellular AA fluxes. In order to elucidate the substrate specificity of CLDN8, the flux rates of AAs were investigated by LC-MS/MS. Glycine was unable to measure below the detection limit under our experimental conditions. The fluxes to AAs, especially middle molecular size AAs including Lys, Glu, His, and Phe, were enhanced by *CLDN8* silencing (Figure 6).

### 3.6. Effect of Aging on Colonic CLDN8 and miR-153-5p Expression

**Figure 7:**
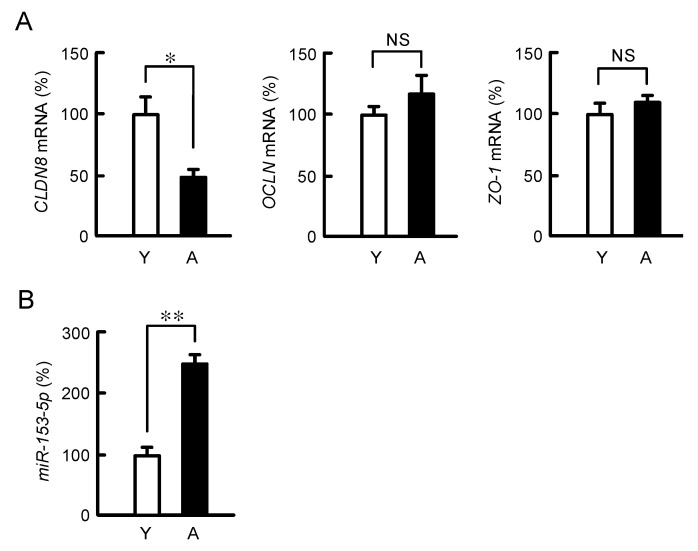
Effect of aging on the expression levels of intestinal TJ components and miR-153-5p. (**A**) The mRNA levels of *CLDN8*, *OCLN*, and *ZO-1* in the colons of aged mice (A, 49 weeks) were shown as a percentage of young mice (Y, 6 weeks). (**B**) The miR-153-5p levels in the colon were shown as a percentage of young mice. *n* = 5. ** *p* < 0.01, * *p* < 0.05 and ^NS^ *p* > 0.05.

**Figure 8:**
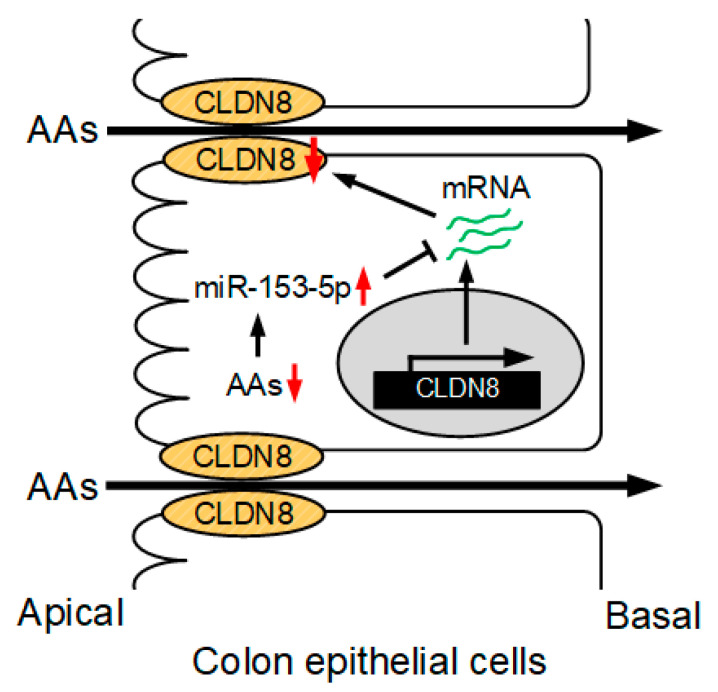
A putative scheme of the regulation of CLDN8 expression in the colon. The reduction in AAs concentration may enhance paracellular AA fluxes from the apical to basolateral chambers mediated by the elevation of miR-153-5p and reduction in CLDN8 expression.

The mRNA level of *CLDN8* in the colon was significantly decreased by aging, whereas the levels of OCLN and ZO-1 were unaffected (Figure 7A). The expression level of miRNA-153-5p in the intestine of aged mice was significantly higher than that of young mice (Figure 7B). These data are consistent with those in the MCE301 cells exposed to AAs deprivation. The scheme of regulation of CLDN8 expression by AAs is depicted in Figure 8.

## 4. Discussion

The barrier function of TJs is determined by the expression pattern of CLDN subtypes [18,19]. Each CLDN subtype is expressed in a tissue-specific manner. In the present study, we found that CLDN8 expression is decreased by AAs deprivation in MCE301 cells (Figure 1 and Figure 2). The CLDN8 expression is upregulated by aldosterone and glucocorticoid signaling pathways in the mouse collecting duct-derived mCCD cells [24] and lung epithelial cells [25], respectively. There are some reports concerning the downregulation of CLDN8 expression in cancer cells by miRNAs including miR-223, miR-361-5p, or miR-340-5p [26,27,28]. The miRNA targets software MIRANDA predicted 14 kinds of miRNAs targeting for the *CLDN8* 3′-untranslated region (Figure 4). The mRNA expression and functional analyses indicated that miR-153-5p may be implicated in the AAs deprivation-induced decline of *CLDN8* expression in MCE301 cells (Figure 4 and Figure 5). The AAs deprivation induces the activation of AMPK/mTOR and autophagy pathways [22]. However, the reduction in CLDN8 expression was not inhibited by AMPK inhibitor, mTOR activator, and autophagy inhibitors (Figure 3C,D). Therefore, our data demonstrate that AMPK/mTOR and autophagy pathways may not be implicated in the AAs deprivation-induced decline of CLDN8 expression. The recovery effect of miR-153-5p inhibitor on the AAs deprivation-induced decline of *CLDN8* expression was partial (Figure 4B). In addition, the reporter activity of CLDN8 was unchanged by AAs deprivation (Figure 3E). These data suggest that the stability of *CLDN8* mRNA may be regulated by other factors. General control non-depressible 2 (GCN2) can be activated by AAs deprivation [29,30], similarly to mTOR. GCN2 is usually implicated in the control of the mRNA translation. Therefore, GCN2 may not participate in the AAs deprivation-induced decline of *CLDN8* expression. We need to conduct further study to unravel the overall regulatory mechanisms of CLDN8 expression.

CLDN8 has been known to function as a cation barrier using Madin-Darby canine kidney cells [31]. TER was increased by AAs deprivation in MCE301 cells, which was blocked by a miR-153-5p inhibitor. In addition, the CLDN8 silencing decreased TER. These results suggest that CLDN8 functions as a barrier to mineral ions in MCE301 cells. The CLDN8 expression and TJs barrier function are attenuated by the infection of human colonic epithelial HT-29/B6 cells to Arcobacter butzleri [32] or Campylobacter concisus [33], which are pathogenic bacteria causing diarrhea. Pigs fed on low-protein diets have decreased incidence of post-weaning diarrhea [34]. It is believed that the improvement effect is caused by the inhibition of proliferation of pathogenic bacteria and the adhesion of enterotoxigenic bacteria to the intestinal epithelial mucosa. However, there is a possibility that TJs barrier function is attenuated by low-protein diets mediated by the reduction in CLDN8 expression. The miR-153-5p inhibitor may be useful to enhance the improvement effect on bacteria-induced diarrhea.

Several CLDNs can also make a barrier to small aqueous molecules. We indicated that the AA fluxes from apical to basal chambers are enhanced by the CLDN8 silencing in MCE301 cells under low-temperature conditions (Figure 5), which can avoid the involvement of AA transporters. The CLDN8 silencing enhanced the fluxes to Lys, Glu, His, and Phe (Figure 6), suggesting that CLDN8 forms a physical barrier to middle molecular size AAs. In comparison to acidic AAs, the flux to Glu was enhanced by the *CLDN8* silencing, but that to Asp was not. In the comparison of basic AAs, Lys, His, and Arg, the fluxes to Lys and His were enhanced by *CLDN8* silencing, but the flux to Arg was not. We suggest that CLDN8 does not form a charge barrier to AAs. So far, Wada et al. [35] have demonstrated that CLDN2 and CLDN15 double knockout mice exhibit a lack of paracellular Na^+^ flow, leading to the reduction in nutrient transports including glucose, AAs, and fats in the small intestine and colon. The double knockout inhibits Na^+^ backleak from the submucosa to the intestinal lumen. Therefore, they suggest that the reduction in nutrient transport is caused by the inhibition of Na^+^-dependent absorption mediated via AA transporters. The absorption rates of the essential AAs including Val, Leu, Iso, Trp, Phe, and Tyr in wild-type mice are higher than those in CLDN2 and CLDN15 double-knockout mice. The absorption rates of most AAs are increased by the addition of Na^+^ in the knockout mice. We suggest that CLDN8 functions as an AAs barrier, and the substrate specificity is different from that of AA transporters. The distribution in the TJs and the function of CLDN4 are controlled by CLDN8 expression in the collecting ducts [36]. Therefore, we cannot deny the involvement of other CLDN subtypes in the regulation of paracellular AA fluxes in the colon.

Healthy physical excise stimulates the metabolic rate of nutrients, including the promotion of protein digestion, AAs oxidation, and suppression of proteosynthesis in humans. Requirements for AAs may also change qualitatively as an animal physically matures. The decline in serum total AAs concentration is related to reduced activation energy with aging [7]. Dietary protein limitation exerts multiple roles including extending lifespan and enhancing stress resistance. In contrast, an imbalanced AAs intake can depress the immune system and increase the susceptibility to infections [37]. Therefore, a low protein intake may be detrimental when applied to older ages [38]. CLDN8 may be a novel target for the prevention of AAs deficiency in older ages.

## 5. Conclusions

We found that the expression of CLDN8 is downregulated by AAs deprivation in MCE301 cells. The AAs deprivation-induced decline of CLDN8 expression was reversed by a miR-153-5p inhibitor. The CLDN8 silencing enhanced the fluxes to AAs, especially middle molecular size AAs. The upregulation of miR-153-5p and downregulation of CLDN8 expression were detected in the colons of aged mice. Our data suggest that CLDN8 functions as a barrier against AAs under normal conditions. The reduced CLDN8 expression may be compensatory to the decreased AAs contents.

## Figures and Tables

**Table 1:** The lists of primary antibodies.

Primary Antibodies	Sources
Rabbit anti-CLDN8 antibody	Thermo Fisher Scientific (Rockford, IL, USA)
Mouse anti-ZO-1 antibody	Thermo Fisher Scientific (Rockford, IL, USA)
Goat anti-β-actin antibody	Santa Cruz Biotechnology (Santa Cruz, CA, USA)
Rabbit anti-phospho-mammalian targets of rapamycin (Ser2448) (p-mTOR)	Cell Signaling Technology (Danvers, MA, USA)
Rabbit anti-mTOR antibody	Cell Signaling Technology (Danvers, MA, USA)
Rabbit anti-AMPK antibody	ProteinTech (Rosemont, IL, USA)
Rabbit anti-phospho-AMPK (T183/T172) (p-AMPK) antibody	R&D Systems (Minneapolis, MN, USA)

**Table 2:** Primer pairs for real-time PCR of TJ components.

Genes	Direction	Sequence (5′→3′)
*CLDN1*	Sense	GTCTTCGATTCCTTGCTGAA
	Antisense	CCTGGCCAAATTCATACCTG
*CLDN2*	Sense	TGCGACACACAGCACAGGCATCAC
	Antisense	TCAGGAACCAGCGGCGAGTAGAA
*CLDN3*	Sense	CATCCTGCTGGCCGCCTTCG
	Antisense	CCTGATGATGGTGTTGGCCGAC
*CLDN8*	Sense	CATGCCAACATCAGAATGCAGT
	Antisense	CTGTGGTCCAGCCTATGTAGAG
*OCLN*	Sense	TGGATCTATGTACGGCTCACAG
	Antisense	AAAGCCACGATAATCATGAACC
*ZO-1*	Sense	CAGAGCCTCAGAAACCTCAAGT
	Antisense	TCTTCGGTCAAAGTAGGAGAGC
*β-Actin*	Sense	CCAACCGTGAAAAGATGACC
	Antisense	CCAGAGGCATACAGGGACAG

**Table 3:** Primers for real-time PCR of miRNAs.

Genes	Sequence (5′→3′)
*miR-134-5p*	TGTGACTGGTTGACCAGAGG
*miR-147-5p*	TGGAAACATTTCTGCACAAAC
*miR-153-5p*	GTCATTTTTGTGACGTTGCAG
*miR-291b-3p*	AAAGTGCATCCATTTTGTTTG
*miR-323-3p*	CACATTACACGGTCGACCTC
*miR-350-5p*	AAAGTGCATGCGCTTTGGG
*miR-466m-3p*	TACATACACACATACACACG
*miR-466o-3p*	TACATACATGCACACATAAG
*miR-1948-3p*	TTTAGGCAGAGCACTCGTAC
*miR-3059-5p*	TTTCCTCTCTGCCCCATAGG
*miR-3108-5p*	GTCTCTAAAGCTAGACGTTC
*miR-5616-5p*	TTTCCTCTCATCACAAGTTG
*miR-7226-3p*	TGACACAGCCATTCTCTGAG
*miR-12206-5p*	TACTATGCCTGGAAGGCACC

**Table 4:** List of monitored [M + H]^+^.

AAs	[M + H]^+^
Phe	166.0918
Trp	205.0967
Leu_Ile	132.1026
Met	150.06
Tyr	182.0836
Pro	116.0715
Val	118.0862
Ala	90.0551
Thr	120.0662
Glu	148.0616
Asp	134.0455
Ser	106.0506
Gln	147.077
Asn	133.0617
(Cys)2	241.0299
His	156.0783
Lys	147.1142
Arg	175.1255

## Data Availability

Not applicable.

## Abbreviations

AA	Amino acid
CLDN	Claudin
DAPI	4′,6-diamidino-2-phenylindole
DMEM	Dulbecco’s modified Eagle’s medium
3MA	3-methyladenine
miRNA	MicroRNA
OCLN	Occludin
p-mTOR	Phospho-mammalian target of rapamycin
PCR	Polymerase chain reaction
TER	Transepithelial electrical resistance
TJ	Tight junction
ZO-1	Zonula occludens-1
